# Supplementary material for: Finite-time quantum entanglement in propagating squeezed microwaves
Source: Sci Rep. 2018 Apr 23;8:6416. doi: 10.1038/s41598-018-24742-z (PMC5913304; doi:10.1038/s41598-018-24742-z)
Supplement: Supplementary file 1 — Supplementary Material [file 41598_2018_24742_MOESM1_ESM.pdf]

Supplementary online material for the paper "Finite-time quantum entanglement in propagating squeezed microwaves"

K. G. Fedorov[1,*] S. Pogorzalek[1,2], U. Las Heras[4], M. Sanz[4], P. Yard[1,2], P. Eder[1,2,3], M. Fischer[1,2,3], J. Goetz[1,2], E. Xie[1,2,3], K. Inomata[7,8], Y. Nakamura[7,9], R. Di Candia[10], E. Solano[2,4,5,6], A. Marx[1], F. Deppe[1,2,3], and R. Gross[1,2,3†]

[1] *Walther-Meißner-Institut, Bayerische Akademie der Wissenschaften, D-85748 Garching, Germany*
[2] *Physik-Department, Technische Universität München, D-85748 Garching, Germany*
[3] *Nanosystems Initiative Munich (NIM), Schellingstraße 4, 80799 München, Germany*
[4] *Department of Physical Chemistry, University of the Basque Country UPV/EHU, Apartado 644, E-48080 Bilbao, Spain*
[5] *IKERBASQUE, Basque Foundation for Science, Maria Diaz de Haro 3, 48013 Bilbao, Spain*
[6] *Department of Physics, Shanghai University, 200444 Shanghai, China*
[7] *RIKEN Center for Emergent Matter Science (CEMS), Wako, Saitama 351-0198, Japan*
[8] *National Institute of Advanced Industrial Science and Technology, 1-1-1 Umezono, Tsukuba, Ibaraki 305-8563, Japan*
[9] *Research Center for Advanced Science and Technology (RCAST), The University of Tokyo, Meguro-ku, Tokyo 153-8904, Japan*
[10] *Freie Universität Berlin, Institut für Theoretische Physik, Arnimallee 14, 14195 Berlin, Germany*

(Dated: March 24, 2018)

SAMPLE DETAILS

The basis of our Josephson parametric amplifier (JPA) consists of a quarter-wavelength superconducting microwave resonator in a coplanar waveguide (CPW) geometry (see Fig. 1, Refs. 1, 2). By short-circuiting the microwave resonator on one side to the ground plane via a direct current superconducting quantum interference device (dc-SQUID), the resonant frequency f_0 of the JPA can be tuned by an external magnetic flux applied to the dc-SQUID loop via an external coil or via an on-chip antenna ("pump line"). The JPA samples used in this work were designed and fabricated at NEC Smart Energy Research Laboratories, Japan and RIKEN, Japan. Thermally oxidized silicon with a thickness of 300 μm is used as a substrate. The resonator and the pump line are patterned into a previously sputtered Nb film with a thickness of 50 nm. A coplanar waveguide (CPW) geometry is used for the resonator and the pump line. The dc-SQUID is fabricated using the aluminum shadow evaporation technique.

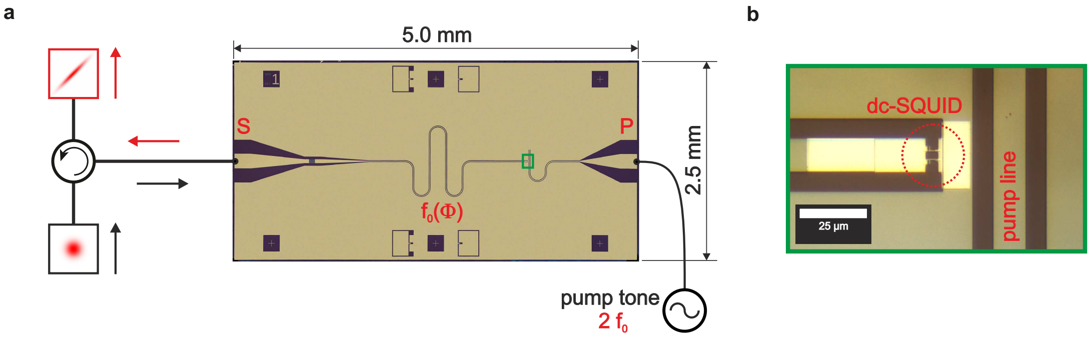

FIG. 1: Part **a** shows the scheme for generation of a squeezed vacuum state and the optical micrographs of the JPA sample chip with the signal line (S) and the pump line (P). The area of the dc-SQUID is marked by the green outline and is shown on the enlarged scale in **b**. The size of the dc-SQUID loop is 4.2 × 2.4 μm^2. Light areas: Al. Dark-brown areas: substrate. Light-brown areas: Nb.

Two JPAs (with the design shown in Fig. 1) have been used for the current experiments of generation of propagating microwave TMS states. Table I summarizes the most relevant parameters of the used JPAs obtained from spectroscopic characterization measurements [3].

The operational principle of squeezing with a flux-driven JPA consists in applying a strong microwave pump tone at port P at the frequency $2f_0$, where f_0 is the flux-tuneable resonant JPA frequency. This results in parametric degenerate (phase-dependent) amplification of an incoming mode f_0 at the signal port. In the case of incoming vacuum (no signal) the JPA effectively produces a squeezed vacuum state which leaks out from port S and can be

TABLE I: Parameters extracted from fitting of the flux-dependent JPA resonant frequency. The external quality factors Q_{ext} and internal quality factors Q_{int} are obtained from independent fits of the JPA spectral linewidths [3]. The critical current I_c is given per Josephson junction, E_J is the corresponding Josephson energy, and β_L denotes the screening parameter of the dc-SQUID.

Sample	I_{c} (μA)	β_{L}	L_{loop} (pH)	$f_0/2\pi$ (GHz)	E_{J}/h (THz)	Q_{ext}	Q_{int}
JPA 1	2.45	0.09	35.8	5.808	1.22	300–360	>30000
JPA 2	2.41	0.10	40.7	5.838	1.20	240–260	>30000

used as a quantum resource for further operations. To separate the incoming (vacuum) and outgoing (squeezed vacuum) signals we employ commercial cryogenic circulators (see Fig. 2).

EXPERIMENTAL SCHEMATICS

The dual-path receiver [1, 4] and the cryogenic double-JPA setup are shown in Fig. 2. Squeezed microwave states are produced by the JPAs, which are stabilized at the temperature of $T = 50$ mK. Then, the resulting states are superimposed by means of a microwave cryogenic hybrid ring beam splitter producing two path-entangled modes containing TMS states. Isolators and circulators provide isolation from spurious reflections. After the first amplification stage with the cryogenic high-electron-mobility transistor (HEMT) amplifiers, the signals are further amplified at room temperature by a chain of rf-amplifiers, which are also temperature-stabilized by a Peltier cooler. Bandpass filters (4.9-6.2 GHz) provide a rough filtering around the desired JPA frequency f_0.

For the data acquisition, we use a two-stage down-conversion scheme as shown in Fig. 2. First, we downconvert the amplified microwave signals to a fixed intermediate frequency (IF) by mixing input signals at f_0 with a detuned strong local oscillator (LO) such that $f_{\text{IF}} = f_{\text{LO}} - f_0 = 11$ MHz. For this mixing process, we use image rejection mixers. The use of these mixers is crucial for filtering out an unwanted signal sideband at frequencies $f_{\text{side}} = f_0 + 22$ MHz, which otherwise contributes to the IF mode and fundamentally limits the reconstructed squeezing levels to 3 dB. We use tunable attenuators for analog pre-balancing of the two channels. Narrow-band filters (9.5-11.5 MHz) further reduce noise and protect the subsequent IF amplifiers from saturation. Finally, an additional chain of IF filters and dc-blocks purges the signals before digitization.

In order to convert the analog signals to the digital domain, we use the Acqiris DC440 analog-to-digital conversion (ADC) card. After transferring the digitized IF data to a computer, we perform a digital downconversion using an efficient, custom-made, multi-threaded acquisition program. The latter also applies fine digital filtering of the incoming signals using a finite-impulse-response (FIR) filter with a tunable bandwidth Ω. The same sofware is used to calculate all correlation moments $\langle I_1^n I_2^m Q_1^k Q_2^l \rangle$ with $n+m+k+l \leq 4$ for $n, m, k, l \in \mathbb{N}$, and to perform averaging. After each averaging cycle, typically consisting of 1.5×10^9 samples, we apply the reference state reconstruction code in order to extract the signal operator moments $\langle (\hat{a}^\dagger)^n \hat{a}^m \rangle$ and build a corresponding Wigner function. The extracted "on the fly" squeezing angles ϕ_{exp1} and ϕ_{exp2} are used to calculate a phase correction $\delta\phi_{1,2} = \phi_{\text{exp1,2}} - \phi_{\text{target1,2}}$ in order to stabilize the squeezing angle by correcting the phase of the microwave pump tone by $\delta\phi_{\text{pump1,2}} = 2\,\delta\phi_{1,2}$. Finally, the described cycle is repeated approximately 50-100 times in order to obtain sufficient statistics.

INPUT-OUTPUT THEORY

For the description of the properties of SMS and TMS states, let us first consider the following Hamiltonian of a nonlinear driven resonator (in our case acts as a JPA) with a fundamental frequency $f_0 = \omega_0 2\pi$:

$$H = H_{free} + i\frac{\chi}{2}(a^2 - a^{\dagger 2}) + i\sqrt{\frac{k}{2\pi}}\int d\omega[b(\omega)a^\dagger - ab^\dagger(\omega)] + i\sqrt{\frac{\gamma}{2\pi}}\int d\omega[c(\omega)a^\dagger - ac^\dagger(\omega)], \quad (1)$$

where

$$H_{free} = \omega_0 a^\dagger a + \int d\omega \omega b^\dagger(\omega)b(\omega) + \int d\omega \omega c^\dagger(\omega)c(\omega) \quad (2)$$

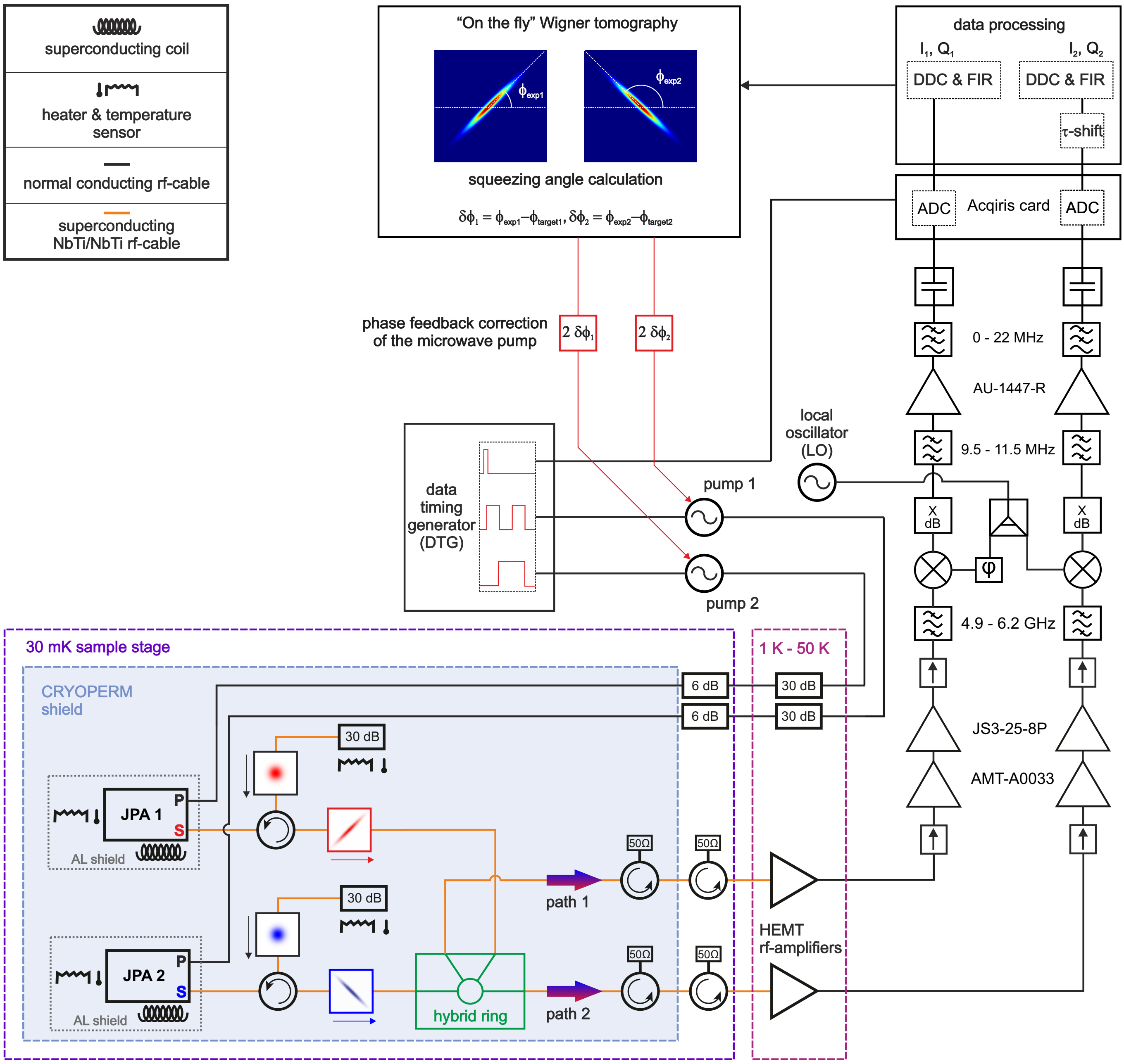

FIG. 2: Scheme of the experimental setup for generation and characterization of propagating SMS and TMS states in the dual-path microwave setup (blue-red arrows mark the propagating entangled signals). A simplified view of the pulsing mechanism by the digital time generator (DTG) is shown. The "DDC & FIR" part in the "data processing" box denotes the digital downconversion and the finite impulse response filtering.

is the free Hamiltonian. The first term in Eq. 1 is the squeezing Hamiltonian with nonlinearity χ, the second term is the coupling between the input signal and the transmission line with a coupling rate κ, while the last part is the coupling with a bath with a coupling rate γ. Here and further, for simplicity, we omit operator hats in all equations and set $\hbar = 1$. Defining $b(\omega) \equiv Be^{-i\omega_0 t}$, $c(\omega) \equiv Ce^{-i\omega_0 t}$, the Heisenberg equations for the modes $B(\omega)$ and $C(\omega)$ in the interaction picture of $H_I = \omega_0 a^\dagger a$ are

$$\dot{B}(\omega) = -i(\omega - \omega_0)B(\omega) - \sqrt{\frac{k}{2\pi}}a. \tag{3}$$

The solution of Eq. (3) can be written in terms of two different initial conditions: with respect the input signal $B_0(\omega)$ at time $t_0 < t$, or with respect the output signal $B_1(\omega)$ at time $t_1 > t$:

$$B(\omega) = e^{-i(\omega-\omega_0)(t-t_0)}B_0(\omega) - \sqrt{\frac{k}{2\pi}}\int_{t_0}^{t} dt' e^{-i(\omega-\omega_0)(t-t')}a(t') \qquad t_0 < t \tag{4}$$

$$B(\omega) = e^{-i(\omega-\omega_0)(t-t_1)}B_1(\omega) + \sqrt{\frac{k}{2\pi}}\int_{t}^{t_1} dt' e^{-i(\omega-\omega_0)(t-t')}a(t') \qquad t_1 > t. \tag{5}$$

Similar equations hold for $c(\omega)$. Regarding the resonator mode a, we have

$$\begin{aligned}
\dot{a} = -i[a,H] &= \chi e^{i2\omega_0 t}a^\dagger + \sqrt{\frac{k}{2\pi}}\int d\omega B(\omega) + \sqrt{\frac{\gamma}{2\pi}}\int d\omega C(\omega) \\
&= \chi e^{i2\omega_0 t}a^\dagger + \sqrt{\frac{k}{2\pi}}\int d\omega e^{-i(\omega-\omega_0)(t-t_0)}B_0(\omega) - \frac{k}{2\pi}\int_{t_0}^{t} dt' \int d\omega e^{-i(\omega-\omega_0)(t-t')}a(t') \\
&\quad + \sqrt{\frac{\gamma}{2\pi}}\int d\omega e^{-i(\omega-\omega_0)(t-t_0)}C_0(\omega) - \frac{\gamma}{2\pi}\int_{t_o}^{t} dt' \int d\omega e^{-i(\omega-\omega_0)(t-t')}a(t') \\
&= \chi e^{i2\omega_0 t}a^\dagger - \frac{k+\gamma}{2}a + \sqrt{k}b_{in} + \sqrt{\gamma}c_{in},
\end{aligned} \tag{6}$$

where we have defined $b_{in}(t) \equiv \frac{1}{\sqrt{2\pi}}\int d\omega e^{-i(\omega-\omega_0)(t-t_0)}B_0(\omega)$, and the same for $c_{in}(t) \equiv \frac{1}{\sqrt{2\pi}}\int d\omega e^{-i(\omega-\omega_0)(t-t_0)}C_0(\omega)$, and we have used the identities $\int d\omega e^{-i\omega(t-t')} = 2\pi\delta(t-t')$, $\int_{t_0}^{t} dt' f(t')\delta(t-t') = \frac{1}{2}f(t)$.

Equation (6) is the dynamical equation for the intra-resonator field, depending on the input signal b_{in} and loss mode c_{in}. If we define the output modes $b_{out}(t) \equiv \frac{1}{\sqrt{2\pi}}\int d\omega e^{-i(\omega-\omega_0)(t-t_1)}b_1(\omega)$ and $c_{out}(t) \equiv \frac{1}{\sqrt{2\pi}}\int d\omega e^{-i(\omega-\omega_0)(t-t_1)}c_1(\omega)$, from Eqs. (4)-(5) (and the equivalent equations for c), we find the boundary conditions

$$b_{out} = b_{in} - \sqrt{k}a \tag{7}$$

$$c_{out} = c_{in} - \sqrt{\gamma}a. \tag{8}$$

SQUEEZING

Here, we derive how much squeezing we have in the steady-state of the output signal. Let us consider the equations for the field quadratures $q_a \equiv (a+a^\dagger)/2$, $p_a \equiv -i(a-a^\dagger)/2$ ($[q_a,p_a] = i/2$, as $[a,a^\dagger]=1$).

$$\dot{q}_a = \left(\chi - \frac{k+\gamma}{2}\right)q_a + \sqrt{k}q_{b_{in}} + \sqrt{\gamma}q_{c_{in}}, \tag{9}$$

$$\dot{p}_a = -\left(\chi + \frac{k+\gamma}{2}\right)p_a + \sqrt{k}p_{b_{in}} + \sqrt{\gamma}p_{c_{in}}, \tag{10}$$

where $q(p)_{b(c)_{in}}$ have been defined similarly as $q(p)_a$. For the steady state, we obtain that $\dot{q}_a = \dot{p}_a = 0$, so

$$q_a = -\frac{2\sqrt{k}}{2\chi-k-\gamma}q_{b_{in}} - \frac{2\sqrt{\gamma}}{2\chi-k-\gamma}q_{c_{in}}, \tag{11}$$

$$p_a = \frac{2\sqrt{k}}{2\chi+k+\gamma}p_{b_{in}} + \frac{2\sqrt{\gamma}}{2\chi+k+\gamma}p_{c_{in}}. \tag{12}$$

Using the boundary conditions, we have that

$$q_{b_{out}} = \frac{2\chi+k-\gamma}{2\chi-k-\gamma}q_{b_{in}} + \frac{2\sqrt{k\gamma}}{2\chi-k-\gamma}q_{c_{in}}, \tag{13}$$

$$p_{b_{out}} = \frac{2\chi-k+\gamma}{2\chi+k+\gamma}p_{b_{in}} - \frac{2\sqrt{k\gamma}}{2\chi+k+\gamma}p_{c_{in}}. \tag{14}$$

Here, the c_{in} corresponds to the coupled noise mode, and it is usually taken to be the vacuum or a thermal state. Note that, in the case $\gamma = 0$, we have perfect squeezing with intensity gain $G_q = \left(\frac{2\chi+k}{2\chi-k}\right)^2 = \frac{1}{G_p}$.

Now, let us solve the dynamical equation (6) using the Fourier transform. If we define $a(t) = \frac{1}{\sqrt{2\pi}} \int d\omega e^{-i\omega_0 t}\tilde{a}(\omega)$ (we move to a frame rotating with ω_0), we have that

$$-i\omega\tilde{a}(\omega) = \chi\tilde{a}^\dagger(-\omega) - \frac{k+\gamma}{2}\tilde{a}(\omega) + \sqrt{k}\tilde{b}_{in}(\omega) + \sqrt{\gamma}\tilde{c}_{in}(\omega). \tag{15}$$

If we define the quadratures $Q_a(\omega) = (\tilde{a}(\omega) + \tilde{a}^\dagger(-\omega))/2$, $P_a(\omega) = -i(\tilde{a}(\omega) - \tilde{a}^\dagger(-\omega))/2$, we have the following squeezing relations for the intraresonator field:

$$Q_a(\omega) = -\frac{\sqrt{k}}{\chi - \frac{k+\gamma}{2} + i\omega} Q_{b_{in}}(\omega) - \frac{\sqrt{\gamma}}{\chi - \frac{k+\gamma}{2} + i\omega} Q_{c_{in}}(\omega) \tag{16}$$

$$P_a(\omega) = \frac{\sqrt{k}}{\chi + \frac{k+\gamma}{2} - i\omega} P_{b_{in}}(\omega) + \frac{\sqrt{\gamma}}{\chi + \frac{k+\gamma}{2} - i\omega} P_{c_{in}}(\omega). \tag{17}$$

Substituting the boundary conditions, we arrive at the relations

$$Q_{b_{out}}(\omega) = \frac{2\chi + k - \gamma + 2i\omega}{2\chi - k - \gamma + 2i\omega} Q_{b_{in}}(\omega) + \frac{2\sqrt{k\gamma}}{2\chi - k - \gamma + 2i\omega} Q_{c_{in}}(\omega) \tag{18}$$

$$P_{b_{out}}(\omega) = \frac{2\chi - k + \gamma - 2i\omega}{2\chi + k + \gamma - 2i\omega} P_{b_{in}}(\omega) - \frac{2\sqrt{k\gamma}}{2\chi + k + \gamma - 2i\omega} Q_{c_{in}}(\omega). \tag{19}$$

NEGATIVITY IN TERMS OF INTERNAL PARAMETERS AND FILTER FUNCTION

We now calculate the negativity for an entangled system in which one of the paths has suffered a time delay. In order to get an expression for the negativity, it is necessary to compute the terms of the covariance matrix. The system we study is composed of two squeezed states with squeezing phases ϕ_1 and ϕ_2, respectively, which are sent to the two ports of an entangling beam splitter that introduces the following operation:

$$a_1' = \frac{1}{\sqrt{2}}(a_1 + a_2), \tag{20}$$

$$a_2' = \frac{1}{\sqrt{2}}(a_1 - a_2), \tag{21}$$

where a_1' and a_2' are the fields emerging from the output ports of the beam splitter. After this, a time delay τ operation is introduced in the second beam. Finally, a measurement is performed in the generalized quadrature $x'_{i,\lambda_i} = (a_i' e^{-i\lambda_i} + a_i'^\dagger e^{i\lambda_i})/2$ of path $i = 1, 2$ with a phase λ_i, where we have to take into account the filter function. Here, λ_i denotes the angle in which we are measuring and which corresponds to quadrature q when $\lambda_i = 0$, or p when $\lambda_i = \pi/2$. Accordingly, in order to calculate the negativity (or negativity kernel), which is a function of the covariance matrix, which elements should be calculated first. These elements are

$$\begin{aligned}\langle x'_{1,\lambda_1}(0) x'_{2,\lambda_2}(\tau)\rangle &= \frac{1}{4}\langle (a_1'(0)e^{-i\lambda_1} + a_1'^\dagger(0)e^{i\lambda_1})(a_2'(\tau)e^{-i\lambda_2} + a_2'^\dagger(\tau)e^{i\lambda_2})\rangle \\ &= \frac{1}{8}\langle\chi_1,\chi_2|\{[a_1(0) + a_2(0)]e^{-i\lambda_1} + [a_1^\dagger(0) + a_2^\dagger(0)]e^{i\lambda_1}\} \\ &\times \{[a_1(\tau) - a_2(\tau)]e^{-i\lambda_2} + [a_1^\dagger(\tau) - a_2^\dagger(\tau)]e^{i\lambda_2}\}|\chi_1,\chi_2\rangle.\end{aligned} \tag{22}$$

We now introduce the transformation of a and $a^\dagger$ operators for squeezed states

$$\hat{S}^\dagger(\xi) a \hat{S}(\xi) = aC - a^\dagger e^{i\phi} S, \tag{23}$$

$$\hat{S}^\dagger(\xi) a^\dagger \hat{S}(\xi) = a^\dagger C - a e^{-i\phi} S, \tag{24}$$

where $\xi = re^{i\phi}$, and $C \equiv \cosh(r)$ and $S \equiv \sinh(r)$. Hence, Eq. 22 yields

$$\begin{aligned}&\frac{1}{8}\langle 0|\{a_1(0)[e^{-i\lambda_1}C_1 - e^{i(\lambda_1-\phi_1)}S_1] + a_1^\dagger(0)[e^{i\lambda_1}C_1 - e^{-i(\lambda_1-\phi_1)}S_1] \\ &+ a_2(0)[e^{-i\lambda_1}C_2 - e^{i(\lambda_1-\phi_2)}S_2] + a_2^\dagger(0)[e^{i\lambda_1}C_2 - e^{-i(\lambda_1-\phi_2)}S_2]\} \\ &\times \{a_1(\tau)[e^{-i\lambda_2}C_1 - e^{i(\lambda_2-\phi_1)}S_1] + a_1^\dagger(\tau)[e^{i\lambda_2}C_1 - e^{-i(\lambda_2-\phi_1)}S_1] \\ &- a_2(\tau)[e^{-i\lambda_2}C_2 - e^{i(\lambda_2-\phi_2)}S_2] - a_2^\dagger(\tau)[e^{i\lambda_2}C_2 - e^{-i(\lambda_2-\phi_2)}S_2]\}|0\rangle.\end{aligned} \tag{25}$$

Since $[a_1(t), a_2^\dagger(t')] = 0$, one gets

$$\frac{1}{8}\langle 0|a_1(0)a_1^\dagger(\tau)[e^{-i\lambda_1}C_1 - e^{i(\lambda_1-\phi_1)}S_1][e^{i\lambda_2}C_1 - e^{-i(\lambda_2-\phi_1)}S_1]$$
$$- a_2(0)a_2^\dagger(\tau)[e^{-i\lambda_1}C_2 - e^{i(\lambda_1-\phi_2)}S_2][e^{i\lambda_2}C_2 - e^{-i(\lambda_2-\phi_2)}S_2]|0\rangle, \tag{26}$$

where $\langle 0|a_i(0)a_i^\dagger(\tau)|0\rangle = \langle 0|[a_i(0), a_i^\dagger(\tau)]|0\rangle = \langle 0|[a(\tau), a_i^\dagger(0)]|0\rangle = \mathrm{sinc}(\Omega\tau)$. So, one finally obtains the expression

$$\langle x'_{1,\lambda_1}(0)x'_{2,\lambda_2}(\tau)\rangle = \frac{\mathrm{sinc}(\Omega\tau)}{8}[e^{-i(\lambda_1-\lambda_2)}(C_1^2 - C_2^2) + e^{i(\lambda_1-\lambda_2)}(S_1^2 - S_2^2)$$
$$- 2\cos(\lambda_1+\lambda_2-\phi_1)C_1S_1 + 2\cos(\lambda_1+\lambda_2-\phi_2)C_2S_2]. \tag{27}$$

Following the same formalism, it is possible to obtain covariance matrix entries for the self-correlations $\langle q_i p_i\rangle$

$$\langle x'_{1,\lambda_1}(0)x'_{1,\lambda_2}(0)\rangle = \frac{1}{8}[e^{-i(\lambda_1-\lambda_2)}(C_1^2 + C_2^2) + e^{i(\lambda_1-\lambda_2)}(S_1^2 + S_2^2)$$
$$- 2\cos(\lambda_1+\lambda_2-\phi_1)C_1S_1 - 2\cos(\lambda_1+\lambda_2-\phi_2)C_2S_2], \tag{28}$$

$$\langle x'_{2,\lambda_1}(\tau)x'_{2,\lambda_2}(\tau)\rangle = \frac{1}{8}[e^{-i(\lambda_1-\lambda_2)}(C_1^2 + C_2^2) + e^{i(\lambda_1-\lambda_2)}(S_1^2 + S_2^2)$$
$$- 2\cos(\lambda_1+\lambda_2-\phi_1)C_1S_1 - 2\cos(\lambda_1+\lambda_2-\phi_2)C_2S_2], \tag{29}$$

where $C_i \equiv \cosh(r_i)$ and $S_i \equiv \sinh(r_i)$. If one, instead of squeezed vacuum states, considers a squeezed thermal state then Eqs. (27)-(29) become, respectively,

$$\langle x'_{1,\lambda_1}(0)x'_{2,\lambda_2}(\tau)\rangle = \frac{\mathrm{sinc}(\Omega\tau)}{8}\Big[[e^{-i(\lambda_1-\lambda_2)}(C_1^2 - C_2^2) + e^{i(\lambda_1-\lambda_2)}(S_1^2 - S_2^2)$$
$$- 2\cos(\lambda_1+\lambda_2-\phi_1)C_1S_1 + 2\cos(\lambda_1+\lambda_2-\phi_2)C_2S_2]$$
$$+ (2n_{\mathrm{th}_1})[\cos(\lambda_1-\lambda_2)(C_1^2+S_1^2) - 2\cos(\lambda_1+\lambda_2-\phi_1)C_1S_1]$$
$$- (2n_{\mathrm{th}_2})[\cos(\lambda_1-\lambda_2)(C_2^2+S_2^2) - 2\cos(\lambda_1+\lambda_2-\phi_2)C_2S_2]\Big], \tag{30}$$

$$\langle x'_{1,\lambda_1}(0)x'_{1,\lambda_2}(0)\rangle = \frac{1}{8}\Big[[e^{-i(\lambda_1-\lambda_2)}(C_1^2 + C_2^2) + e^{i(\lambda_1-\lambda_2)}(S_1^2 + S_2^2)$$
$$- 2\cos(\lambda_1+\lambda_2-\phi_1)C_1S_1 - 2\cos(\lambda_1+\lambda_2-\phi_2)C_2S_2]$$
$$+ (2n_{\mathrm{th}_1})[\cos(\lambda_1-\lambda_2)(C_1^2+S_1^2) - 2\cos(\lambda_1+\lambda_2-\phi_1)C_1S_1]$$
$$+ (2n_{\mathrm{th}_2})[\cos(\lambda_1-\lambda_2)(C_2^2+S_2^2) - 2\cos(\lambda_1+\lambda_2-\phi_2)C_2S_2]\Big], \tag{31}$$

$$\langle x'_{2,\lambda_1}(\tau)x'_{2,\lambda_2}(\tau)\rangle = \frac{1}{8}\Big[[e^{-i(\lambda_1-\lambda_2)}(C_1^2 + C_2^2) + e^{i(\lambda_1-\lambda_2)}(S_1^2 + S_2^2)$$
$$- 2\cos(\lambda_1+\lambda_2-\phi_1)C_1S_1 - 2\cos(\lambda_1+\lambda_2-\phi_2)C_2S_2]$$
$$+ (2n_{\mathrm{th}_1})[\cos(\lambda_1-\lambda_2)(C_1^2+S_1^2) - 2\cos(\lambda_1+\lambda_2-\phi_1)C_1S_1]$$
$$+ (2n_{\mathrm{th}_2})[\cos(\lambda_1-\lambda_2)(C_2^2+S_2^2) - 2\cos(\lambda_1+\lambda_2-\phi_2)C_2S_2]\Big], \tag{32}$$

where n_{th_1} and n_{th_2} are average number of thermal photons referred to the inputs of JPA1 and JPA2 respectively. Notice that for this, we have calculated the terms

$$\langle n|a^\dagger(0)a(\tau)|m\rangle = n\ \mathrm{sinc}(\Omega\tau)\delta_{n,m}, \tag{33}$$

$$\langle n|a(0)a^\dagger(\tau)|m\rangle = (n+1)\mathrm{sinc}(\Omega\tau)\delta_{n,m}. \tag{34}$$

We further simplify the covariance matrix terms in Eqs. (30)-(32) to a more convenient form

$$\begin{aligned}\langle x'_{1,\lambda_1}(0)x'_{2,\lambda_2}(\tau)\rangle = \frac{\mathrm{sinc}(\Omega\tau)}{8}\Big[&(1+2n_{\mathrm{th}_1})[\cos(\lambda_1-\lambda_2)(C_1^2+S_1^2)-2\cos(\lambda_1+\lambda_2-\phi_1)C_1S_1]\\ &-(1+2n_{\mathrm{th}_2})[\cos(\lambda_1-\lambda_2)(C_2^2+S_2^2)-2\cos(\lambda_1+\lambda_2-\phi_2)C_2S_2]\Big],\end{aligned} \tag{35}$$

$$\begin{aligned}\frac{1}{2}(\langle x'_{1,\lambda_1}(0)x'_{1,\lambda_2}(0)\rangle + \langle x'_{1,\lambda_2}(0)x'_{1,\lambda_1}(0)\rangle) &= \frac{1}{2}(\langle x'_{2,\lambda_1}(\tau)x'_{2,\lambda_2}(\tau)\rangle + \langle x'_{2,\lambda_2}(\tau)x'_{2,\lambda_1}(\tau)\rangle)\\ &= \frac{1}{8}\Big[(1+2n_{\mathrm{th}_1})[\cos(\lambda_1-\lambda_2)(C_1^2+S_1^2)-2\cos(\lambda_1+\lambda_2-\phi_1)C_1S_1]\\ &\quad+(1+2n_{\mathrm{th}_2})[\cos(\lambda_1-\lambda_2)(C_2^2+S_2^2)-2\cos(\lambda_1+\lambda_2-\phi_2)C_2S_2]\Big].\end{aligned} \tag{36}$$

Notice that $x'_{1,\lambda_1}(0)$ and $x'_{2,\lambda_2}(\tau)$ commute since they act in different subspaces, so there is no need to calculate the term $\langle x'_{2,\lambda_2}(\tau)x'_{1,\lambda_1}(0)\rangle$ as in Eq. (36).

Once the covariance matrix is calculated, one just has to follow the formalism described in Refs. [1, 6]. The covariance matrix here is defined in terms of the quadratures as follows for squeezed but not displaced states

$$\begin{aligned}\sigma &= \begin{pmatrix} \alpha & \gamma \\ \gamma^T & \beta \end{pmatrix}\\ &= \begin{pmatrix} \langle q_1'^2\rangle & \frac{1}{2}\langle q_1'p_1'+p_1'q_1'\rangle & \langle q_1'q_2'\rangle & \langle q_1'p_2'\rangle \\ \frac{1}{2}\langle q_1'p_1'+p_1'q_1'\rangle & \langle p_1'^2\rangle & \langle p_1'q_2'\rangle & \langle p_1'p_2'\rangle \\ \langle q_1'q_2'\rangle & \langle p_1'q_2'\rangle & \langle q_2'^2\rangle & \frac{1}{2}\langle q_2'p_2'+p_2'q_2'\rangle \\ \langle q_1'p_2'\rangle & \langle p_1'p_2'\rangle & \frac{1}{2}\langle q_2'p_2'+p_2'q_2'\rangle & \langle p_2'^2\rangle \end{pmatrix}.\end{aligned} \tag{37}$$

The negativity is defined as

$$\mathcal{N} = \max\left\{0, \frac{1-\nu}{2\nu}\right\}, \tag{38}$$

where $\nu \equiv \frac{1}{\sqrt{2}}\sqrt{\Delta(\sigma)-\sqrt{\Delta^2(\sigma)-4\det\sigma}}$ and $\Delta(\sigma)\equiv \det\alpha+\det\beta-2\det\gamma$. In our case,

$$\begin{aligned}N_k = \frac{1-\nu}{2\nu} = -\frac{1}{2}+\frac{1}{\sqrt{2}}\Big[&1+2n_{\mathrm{th}_1}(1+n_{\mathrm{th}_1})+2n_{\mathrm{th}_2}(1+n_{\mathrm{th}_2})\\ &+(1+2n_{\mathrm{th}_1})(1+2n_{\mathrm{th}_2})\cosh(2(r_1+r_2))+\big(-1-2n_{\mathrm{th}_1}(1+n_{\mathrm{th}_1})-2n_{\mathrm{th}_2}(1+n_{\mathrm{th}_2})\\ &+(1+2n_{\mathrm{th}_1})(1+2n_{\mathrm{th}_2})\cosh(2(r_1+r_2))\big)\mathrm{sinc}(\Omega\tau)^2\\ &-2(1+2n_{\mathrm{th}_1})(1+2n_{\mathrm{th}_2})\sinh(2(r_1+r_2))|\mathrm{sinc}(\Omega\tau)|\Big]^{-1/2}.\end{aligned} \tag{39}$$

Considering $n_1=n_2=0$ and $r_1=r_2=r$, one recovers the negativity for the two-mode squeezed vacuum states with the same squeezing parameters

$$N_k = \frac{1-\nu}{2\nu} = -\frac{1}{2}+\frac{1}{2}\Big(\cosh(2r)^2+\mathrm{sinc}(\Omega\tau)^2\sinh(2r)^2-|\mathrm{sinc}(\Omega\tau)|\sinh(4r)\Big)^{-1/2}. \tag{40}$$

$g^{(2)}(\tau)$ FUNCTION IN TERMS OF INTERNAL PARAMETERS AND FILTER FUNCTION

In the following we derive an analytic expression of the $g^{(2)}(\tau) = \langle\hat{a}^\dagger(0)\hat{a}^\dagger(\tau)\hat{a}(\tau)\hat{a}(0)\rangle/\langle\hat{a}^\dagger(0)\hat{a}(0)\rangle^2$ function for a Gaussian state. For this, we follow the approach of Grosse *et al.* [5], Eq. (8), in which $g^{(2)}(\tau)$ is written as a function of the variances for a Gaussian state. The concrete expression for a squeezed state is:

$$g^{(2)}(\tau) = 1+\mathrm{sinc}^2(\Omega\tau)\frac{1+4\sigma_{\mathrm{s}}^2(2\sigma_{\mathrm{s}}^2-1)+4\sigma_{\mathrm{a}}^2(2\sigma_{\mathrm{a}}^2-1)}{(1-2\sigma_{\mathrm{s}}^2-2\sigma_{\mathrm{a}}^2)^2}, \tag{41}$$

From this equation, one can easily see that the temporal behavior of the $g^{(2)}$ function depends only on the filter, and considering no filter, the time correlation would be constant for any τ. In the opposite case, with a finite bandwidth

filter, we would have a maximal $g^{(2)}$ at $\tau = 0$. Considering a state whose parameters obey Eqs. (13) and (14) the variances are:

$$\sigma_q^2 = \frac{1}{(2\chi-\kappa-\gamma)^2}[(2\chi+\kappa-\gamma)^2\langle q_{b_{in}}^2\rangle + 4\kappa\gamma\langle q_{c_{in}}^2\rangle],$$

$$= \frac{1}{2(2\chi-\kappa-\gamma)^2}[(2\chi+\kappa-\gamma)^2(n_{b_{in}}+1/2) + 4\kappa\gamma(n_{c_{in}}+1/2)], \tag{42}$$

$$\sigma_p^2 = \frac{1}{(2\chi+\kappa+\gamma)^2}[(2\chi-\kappa+\gamma)^2\langle p_{b_{in}}^2\rangle + 4\kappa\gamma\langle p_{c_{in}}^2\rangle],$$

$$= \frac{1}{2(2\chi+\kappa+\gamma)^2}[(2\chi-\kappa+\gamma)^2(n_{b_{in}}+1/2) + 4\kappa\gamma(n_{c_{in}}+1/2)], \tag{43}$$

$$\sigma_{qp} = \frac{\langle q_i p_i + p_i q_i\rangle}{2} = \frac{(2\chi-\kappa+\gamma)(2\chi+\kappa+\gamma)}{(2\chi-\kappa-\gamma)(2\chi+\kappa+\gamma)}\langle q_{b_{in}}p_{b_{in}} + p_{b_{in}}q_{b_{in}}\rangle$$

$$- \frac{2\kappa\gamma}{(2\chi-\kappa-\gamma)(2\chi+\kappa+\gamma)}\langle q_{c_{in}}p_{c_{in}} + p_{c_{in}}q_{c_{in}}\rangle = 0. \tag{44}$$

In the case of squeezed thermal states we can, based on the derivation of Eq. (36), write the covariance matrix entries of a single-mode squeezed state as

$$\sigma_q^2 = \frac{1+2n_{\text{th}}}{4}(C^2+S^2-2CS\cos(\phi)), \tag{45}$$

$$\sigma_p^2 = \frac{1+2n_{\text{th}}}{4}(C^2+S^2+2CS\cos(\phi)), \tag{46}$$

$$\sigma_{qp} = \frac{\langle qp+pq\rangle}{2} = -\frac{1+2n_{\text{th}}}{4}2CS\sin(\phi). \tag{47}$$

For simplicity assuming $\phi_i = 0$ one arrives at:

$$\sigma_q^2 = \sigma_s^2 = \frac{1+2n_{\text{th}}}{4}\exp(-2r), \tag{48}$$

$$\sigma_p^2 = \sigma_a^2 = \frac{1+2n_{\text{th}}}{4}\exp(2r), \tag{49}$$

where σ_s^2 and σ_a^2 are the squeezed and antisqueezed quadrature variances in terms of the squeezing factor r and number of noise photons n_{th}.

* Electronic address: kirill.fedorov@wmi.badw.de
† Electronic address: rudolf.gross@wmi.badw.de

[1] E. P. Menzel, R. Di Candia, F. Deppe, P. Eder, L. Zhong, M. Ihmig, M. Haeberlein, A. Baust, E. Hoffmann, D. Ballester, K. Inomata, T. Yamamoto, Y. Nakamura, E. Solano, A. Marx, and R. Gross, Phys. Rev. Lett. **109**, 250502 (2012).
[2] L. Zhong *et al.*, New J. Phys. **15**, 125013 (2013).
[3] S. Pogorzalek, K. G. Fedorov, L. Zhong, J. Goetz, F. Wulschner, M. Fischer, P. Eder, E. Xie, K. Inomata, T. Yamamoto, Y. Nakamura, A. Marx, F. Deppe, and R. Gross, Phys. Rev. Appl. **8**, 024012 (2017).
[4] E. P. Menzel, F. Deppe, M. Mariantoni, M. A. Araque Caballero, A. Baust, T. Niemczyk, E. Hoffmann, A.Marx, E. Solano, and R. Gross, Phys. Rev. Lett. **105**, 100401 (2010).
[5] N. B. Grosse, T. Symul, M. Stobinska, T. C. Ralph, and P. K. Lam, Phys. Rev. Lett. **98**, 153603 (2007).
[6] G. Adesso and F. Illuminati, Phys. Rev. A **72**, 032334 (2005).
